# Clinical Predictive Factors for the Development of Short Bowel Syndrome in a Cohort of Patients with Crohn’s Disease: A Prospective Study

**DOI:** 10.3390/jcm14176337

**Published:** 2025-09-08

**Authors:** Laura Parisio, Angelo Del Gaudio, Jacopo Iaccarino, Pierluigi Puca, Guia Becherucci, Gaetano Coppola, Carlo Covello, Federica Di Vincenzo, Elisa Foscarini, Lucrezia Laterza, Letizia Masi, Marco Pizzoferrato, Francesca Profeta, Daniela Pugliese, Valentina Petito, Marcello Chieppa, Giammarco Mocci, Giovanni Cammarota, Antonio Gasbarrini, Loris Riccardo Lopetuso, Marcello Covino, Franco Scaldaferri, Alfredo Papa

**Affiliations:** 1 Gastroenterology Unit, Ospedale Isola Tiberina—Gemelli Isola, 00186 Rome, Italy; laura.parisio.fw@fbf-isola.it (L.P.); daniela.pugliese@policlinicogemelli.it (D.P.); 2 Centre for Digestive Diseases (CEMAD) and Gastroenterology Unit, Fondazione Policlinico Universitario A. Gemelli, IRCCS, L.go A. Gemelli, 8, 00168 Rome, Italy; angelo.delgaudio@guest.policlinicogemelli.it (A.D.G.); jacopo.iaccarino01@icatt.it (J.I.); pierluigi.puca@unicatt.it (P.P.); guia.becherucci@policlinicogemelli.it (G.B.); gaetano.coppola@guest.policlinicogemelli.it (G.C.); carlo.covello01@icatt.it (C.C.); federica.divincenzo@guest.policlinicogemelli.it (F.D.V.); elisa.foscarini@policlinicogemelli.it (E.F.); lucrezia.laterza@policlinicogemelli.it (L.L.); letizia.masi@policlinicogemelli.it (L.M.); marco.pizzoferrato@policlinicogemelli.it (M.P.); francesca.profeta@policlinicogemelli.it (F.P.); valentina.petito@policlinicogemelli.it (V.P.); giovanni.cammarota@policlinicogemelli.it (G.C.); antonio.gasbarrini@policlinicogemelli.it (A.G.); franco.scaldaferri@policlinicogemelli.it (F.S.); 3 Dipartimento di Medicina e Chirugia Translazionale, Università Cattolica del S. Cuore, 00168 Rome, Italy; marcello.covino@policlinicogemelli.it; 4 Department of Experimental Medicine (DIMeS), University of Salento, 73100 Lecce, Italy; marcello.chieppa@unisalento.it; 5 Struttura Complessa di Gastroenterologia, Azienda di Rilievo Nazionale ad Alta Specializzazione (ARNAS) G. Brotzù, 09047 Cagliari, Italy; giammarco.mocci@gmail.com; 6 Department of Medicine and Ageing Sciences, G. d’Annunzio University of Chieti-Pescara, 66013 Chieti, Italy; lopetusoloris@libero.it; 7 Center for Advanced Studies and Technology (CAST), G. d’Annunzio University of Chieti-Pescara, 66013 Chieti, Italy; 8 Department of Life Sciences, Health, and Health Professions, Link Campus University, 00165 Rome, Italy; 9 Emergency Medicine, Fondazione Policlinico Universitario A. Gemelli, IRCCS, 00168 Rome, Italy

**Keywords:** short bowel syndrome, Crohn’s disease, intestinal failure, intestinal resection, anti-TNF-α, biological therapy, small molecules

## Abstract

**Background/Objectives**: Crohn’s disease (CD) is one of the most frequent causes of short bowel syndrome (SBS), a severe clinical condition with huge morbidity and social costs. SBS occurs when, following intestinal resections, the remaining small bowel in continuity is less than 200 cm in length. Intestinal failure (IF) can complicate SBS when intravenous nutritional or electrolyte supplementation is required to maintain dietary needs. The primary aim of this study was to identify clinical predictive factors of SBS in a cohort of outpatients with CD. **Methods**: We conducted a prospective, single-center, cohort study enrolling consecutive CD outpatients at a tertiary-level inflammatory bowel disease center. Detailed demographic and clinical features were collected. Significant factors associated with the onset of SBS in the univariate analysis were input into a multivariate logistic regression model to identify independent predictors of SBS. **Results**: In total, 232 CD patients (52.6% male, median age 49 years [IQR 37–60]) were included: 24.6% of them were smokers; extraintestinal manifestations (EIMs) were present in 21.6% of patients; and 67.7% of patients had at least one intestinal resection (27% of them with more than one surgical intervention). At enrollment, 96.1% of patients were on advanced therapies, and considering the course of the disease, 24.6% of patients were exposed to ≥3 different advanced therapies. A total of 18 patients had SBS and 9 had IF. In univariate analysis, the following variables were statistically associated with the risk of developing SBS: disease duration (*p* < 0.001), upper gastrointestinal disease localization (L4) (*p* < 0.001), penetrating behavior (*p* = 0.023), perianal disease (*p* = 0.036), length of first intestinal resection (*p* < 0.001), shorter time elapsing from CD diagnosis to start the first advanced therapy (*p* < 0.001), and treatment with advanced therapy after first intestinal resection (*p* < 0.001). In multivariate analysis, disease duration (OR 1.083, 95% C.I. 1.025–1.145, *p* = 0.005) and L4 (OR 20.079, 95% C.I. 2.473–163.06, *p* = 0.005) were independently associated with the development of SBS. Conversely, the number of different advanced therapies before the onset of SBS was independently associated with a reduced risk of developing SBS (OR 0.247, 95% C.I. 0.107–0.58, *p* = 0.001). **Conclusions**: Our data identifies several clinical features that could possibly predict the development of SBS in CD. Further studies with a larger sample size are needed to confirm our findings.

## 1. Introduction

Crohn’s disease (CD) is a chronic inflammatory bowel disease with progressive stricturing and/or penetrating behavior and an often-destructive course [1].

One of the most fearsome complications of CD is short bowel syndrome (SBS). It occurs following extensive intestinal resections, resulting in a small bowel length of less than 200 cm [2]. The clinical impact of SBS extends beyond malabsorption. Recurrent dehydration, electrolyte disturbances, and micronutrient deficiencies often occur, significantly impairing quality of life. SBS can evolve into intestinal failure (IF) and into chronic intestinal failure (CIF) when intestinal absorption of nutrients and/or electrolytes falls below the threshold necessary to maintain body homeostasis [2].

In addition to SBS, other physiopathological mechanisms can contribute to determining CIF in patients with CD, even in the absence of massive anatomical loss, including extensive mucosal disease, strictures, enterocutaneous fistulas, high-flow stomas, abscesses, or abdominal septic conditions [3]. SBS and CIF have been included in the European Orphanet list of rare diseases [ORPHA:95427 for secondary short bowel syndrome; ORPHA:294422 for chronic intestinal failure] [4,5].

The prevalence of SBS in the general population is estimated to be between 0.4 and 4 cases per million, with CD being the leading cause in adults, accounting for up to 10–20% of cases [6]. However, its true prevalence may be underestimated due to under-recognition in non-specialist settings. Diagnostic underestimation was witnessed by an Italian multicenter survey, which estimated a median prevalence of CIF among CD patients at 1%. The prevalence was significantly higher in academic centers (2.0% [IQR 1–5%] vs. 0.13% [IQR 0–1%], *p* = 0.02) [7].

Recently, higher prevalences of SBS and CIF in CD patients, ranging from 4.2% to 8.6%, were reported from high-volume centers in France [8] and Japan [9].

CD, SBS, and CIF could share several risk factors. Smoking has been identified as a risk factor associated with severe CD and a high risk of surgery, as well as an increased risk of postoperative clinical and surgical recurrence [10]. Patients with a history of smoking were shown to be more likely to develop IF [11]. Beyond smoking, other factors were found to be associated with surgical complications and, consequently, with a higher risk of recurrent surgeries that could predispose to SBS/CIF: preoperative use of systemic steroid treatment, recent weight loss, and intra-abdominal abscess [11,12]. Interestingly, Vaillant et al. found, in a retrospective study involving 410 patients with CD, that those with Montreal B1 (inflammatory behavior) and those treated with budesonide were at a lower risk of SBS, whereas patients treated with IV steroids were at a higher risk [8]. Age at diagnosis below 16 years and a family history of IBD were also associated with a significantly higher risk for developing IF [10]. Moreover, a Japanese retrospective study based on 162 CD patients found that cumulative inflammation, in addition to short residual small intestinal length and non-use of anti-tumor necrosis factor-α therapy, was a potential risk factor for IF in CD [9].

Additionally, gut dysbiosis may play a role in SBS, as it is a crucial factor involved in IBD pathogenesis. An unbalanced gut microbiome has been identified in patients requiring surgical interventions [13]. Therefore, the study of gut microbiota imbalance in CD patients is undoubtedly promising for identifying prognostic markers of SBS and intestinal failure.

Since the early 2000s, the introduction of biological therapies, and more recently small molecules, has raised the hope that better control of CD activity might reduce the need for surgery and, consequently, the incidence of SBS and CIF. However, available data indicate that the widespread use of biologics has not yet resulted in a substantial long-term reduction in surgical rates or severe complications, such as SBS [14]. On the other hand, therapeutic advances, such as the glucagon-like peptide (GLP)-2 analog teduglutide, have proven beneficial by reducing parenteral nutrition requirements in patients with CD and SBS-CIF [15].

Early and reliable prediction of who will progress to SBS is crucial for timely intervention, optimizing nutritional support, and informing surgical decision-making. Currently, prediction is often based on parameters which do not capture the complexity of disease phenotype or the interplay between surgical and clinical factors. This leads to uncertainty in prognosis, delayed referral to intestinal rehabilitation programs, and missed opportunities for preventive strategies. Despite the recognition of the importance of both disease phenotype and surgical metrics, no validated risk prediction tool integrates CD classification with operative variables, such as the extent and location of resection, the number of surgeries, and the preservation of key functional segments. Existing studies do not examine these factors within a unified, reproducible model. This gap limits clinicians’ ability to stratify patients accurately at the time of surgery.

Indeed, given the substantial morbidity and healthcare burden associated with SBS and CIF, identifying modifiable and non-modifiable predictors is crucial for implementing preventive strategies. Therefore, this study aims to identify the risk factors for SBS and CIF, with the goal of implementing effective preventive strategies.

## 2. Materials and Methods

### 2.1. Study Population

This prospective cohort study enrolled consecutive outpatients with CD admitted to our IBD tertiary center from 1 June 2023 to 31 May 2024. The inclusion criteria were age ≥ 18 years, the ability to provide written informed consent, and a definitive diagnosis of CD based on the European Crohn’s and Colitis Organisation (ECCO) criteria [16]. Exclusion criteria included the following: age < 18 years, individuals unable or unwilling to provide informed consent, or those under guardianship without a legally authorized representative available, and a not-defined diagnosis of CD or a diagnosis of undetermined IBD. We excluded patients under 18 years of age because the pediatric gastroenterology unit at our hospital did not participate in the study.

SBS was defined as a residual small bowel length of less than 200 cm, as reported in the surgical reports or through imaging (computed tomography scan or magnetic resonance imaging). CIF was defined according to ESPEN criteria as the need for intravenous supplementation of fluids and/or nutrients for at least 3 days per week for 12 consecutive months or more [17]. Patients with high-output stomas were classified as having CIF only if they met the above-reported criteria for long-term parenteral support.

Exhaustive demographic and clinical data, including previous and current therapies or surgeries, were collected and integrated with information in the patient’s electronic hospital records. In detail, the following variables were assessed: features included in the Montreal classification of CD, smoking, extra-intestinal manifestations (EIM), previous or current medical therapies, including conventional therapy and biologics (anti-tumor necrosis factor [TNF]-α agents, vedolizumab, ustekinumab, anti-IL-23 agents) or small molecules (upadacitinib). Additionally, the number and extent of intestinal resections, as well as the timing of advanced therapy introduction, were recorded.

### 2.2. Study Outcomes

The primary outcome of this study was to identify independent clinical predictors of SBS. The secondary outcome was to determine clinical predictors of CIF.

### 2.3. Statistical Analysis

Continuous variables are presented as medians (interquartile ranges [IQRs]) and compared using the Mann–Whitney U test (for two-group comparisons) and the Kruskal–Wallis ANOVA test (for three-or-more-group comparisons). Categorical variables are indicated in numbers (percentages) and compared using the Chi-squared test or Fisher’s exact test as appropriate. The study variables significantly associated with SBS in univariate analysis were entered into a multivariate logistic regression model to identify independent predictors of SBS.

To avoid model instability, we excluded variables that exceeded the five thresholds for variance inflation due to multicollinearity. In this way, we also aimed to reduce the degrees of freedom of the final logistic model, given the reduced number of events in the study sample.

Logistic regression results are presented as odds ratios (ORs) with 95% confidence intervals (CIs).

Receiver Operating Characteristic (ROC) analysis was used to identify the most accurate threshold for predicting SBS, based on the total number of surgical resections and the extent of resected bowel (measured in cm). The value was chosen according to the Youden index J.

A two-sided *p*-value of ≤0.05 indicated significance.

Since SBS and CIF are rare diseases, as previously reported, we realistically considered including 15–20 patients with SBS with or without CIF. In this context, given an expected prevalence of SBS/CIF of 4.2–8.6% in previous studies [8,9], we would have needed approximately 220–240 patients with CD to enroll.

The Statistical Package for the Social Sciences, version 25 (IBM, Armonk, NY, USA), was used for all data analyses.

## 3. Results

### 3.1. Clinical Characteristics of the Whole Cohort of Patients with CD

We enrolled a total of 277 patients; however, 8 patients were subsequently excluded due to a reformulation of diagnosis (indeterminate colitis or ulcerative colitis), 30 due to a lack of precise information regarding the length of the remaining intestine, and 7 because of withdrawn informed consent.

Finally, a total of 232 patients were included: most patients were male (52.6%), with a median age of 49 years (IQR, 37–60); approximately a quarter of patients were active smokers (24.6%). Other clinical and demographic characteristics are reported in Table 1. Within the cohort, 18 (7.8%) patients were diagnosed with SBS (CD-SBS); in 9 of these, SBS was associated with CIF. Two patients with CIF were on teduglutide treatment. Eight patients had a stoma (3.4%); of these, two had SBS. None of the patients with a stoma had CIF due to a high-output stoma. In the entire cohort, 96.1% of patients were in treatment with an advanced therapy: of these, 111 (47.8%) were on a TNF-α inhibitor, 65 (28%) were on Ustekinumab, and the others were on vedolizumab (*n* = 28, 12.1%), risankizumab (*n* = 15, 6.5%), and upadacitinib (*n* = 4, 1.7%). In total, 57 patients (24.6%) had received three or more different advanced therapies during the disease, and 157 (67.8%) underwent at least one bowel resection.

### 3.2. Comparison of CD Patients with and Without SBS

Comparing patients with SBS with the rest of the population, no significant differences were observed in smoking status, age, or sex (*p* = 0.794; 0.120; 0.793, respectively) (Table 2). At baseline, median C-reactive protein (CRP) levels did not differ between patients with and without SBS [median 4 (IQR 1–9) vs. 3 (IQR 2–8) mg/L, *p* = 0.810].

Patients with SBS had a significantly lower BMI (median 22.3 vs. 23.5, *p* = 0.028) and a longer disease duration (median 27 years vs. 10 years, *p* < 0.001). Upper gastrointestinal involvement (UGI) (L4) was significantly more frequent in SBS patients compared to all other disease localizations (L1 + L2 + L3) (27.8% vs. 3.2%, *p* < 0.001). Similarly, a fistulizing phenotype (B3) was significantly associated with SBS (50% vs. 25.2%, *p* = 0.023). Perianal disease was also more prevalent in CD-SBS patients (44.4% vs. 22.4%, *p* = 0.036), while extraintestinal manifestations did not differ between groups (11.1% vs. 22.4%, *p* = 0.262). The extent of the first intestinal resection was significantly greater (median 100 cm vs. 30 cm, *p* < 0.001) for CD-SBS patients, and, as expected, multiple surgeries (≥3 resections) were significantly more common (44.4% vs. 22%) in this group. Moreover, the age at first surgery was lower in the CD-SBS group, although this difference did not reach statistical significance (median, 31.5 years vs. 37.5 years, *p* = 0.070).

Interestingly, the time from diagnosis to initiation of the first advanced therapy was significantly shorter in CD without SBS than in CD-SBS patients (median, 3.5 years vs. 16 years, *p* < 0.001). Additionally, only 44.4% of CD-SBS patients received biologic therapy after their first surgery, compared to 84.9% of CD patients without SBS (*p* < 0.001).

### 3.3. Focus on Patients with SBS and Intestinal Failure

Comparing patients with CIF to the CD population without IF, we observed that the former had a younger age at diagnosis (median, 33.5 years vs. 37 years, *p* = 0.026) and a longer disease duration (median, 40 years vs. 11 years, *p* = 0.001). UGI (L4) was also significantly more frequent in IF patients compared to all other disease locations (44.4% vs. 3.5%, *p* = 0.001). Notably, a fistulizing disease phenotype (B3) was significantly associated with IF (77.7% vs. 25.1%, *p* = 0.006). The extent of the first intestinal resection was greater in patients with CIF (median 60 cm vs. 30 cm, *p* = 0.027). These data are similar to those observed in patients with SBS. When considering only patients with SBS, no significant differences emerged between those with and without CIF.

Considering the limited number of patients with CIF, a multivariate analysis was not performed.

### 3.4. Multivariate Analysis of Factors Associated with SBS

**Figure 1:**
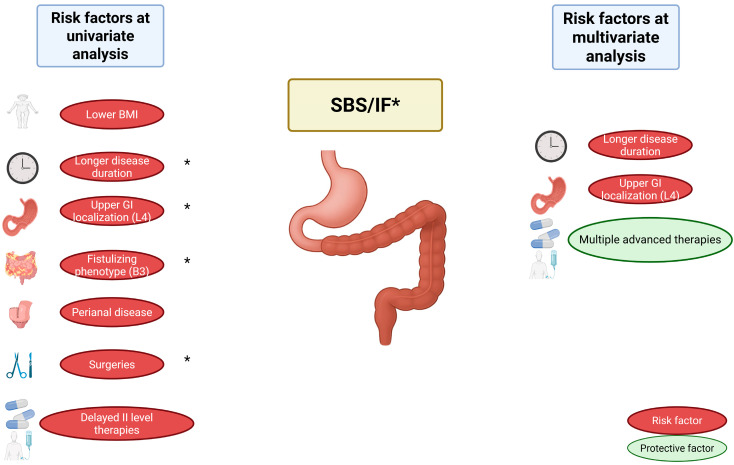
Predictive clinical factors for the development of SBS and CIF in IBD. Risk factors identified at univariate analysis include lower BMI, longer disease duration, upper GI localization (L4), fistulizing phenotype (B3), perianal disease, extent of first surgery, and delayed initiation of advanced (level II) therapies (also after first surgery). At multivariate analysis, longer disease duration and upper GI localization (L4) remained independent predictors, while multiple advanced therapies were identified as a protective factor. Factors marked with an asterisk (*) were also associated with IF. Abbreviations: SBS, Short Bowel Syndrome; IF, Intestinal Failure; IBD, Inflammatory Bowel Disease; GI, Gastrointestinal; BMI, Body Mass Index.

During multivariate logistic regression, three variables were independently associated with the development of SBS (Table 3): upper gastrointestinal involvement (Montreal L4) was strongly associated with an increased risk of SBS (OR 20.08; 95% CI: 2.47–163.06; *p* = 0.005); disease duration also remained significantly associated with SBS (OR 1.08 per year; 95% CI: 1.03–1.15; *p* = 0.005). Conversely, exposure to a higher number of advanced therapies before the onset of SBS was independently associated with a reduced risk of developing SBS (OR 0.25; 95% CI: 0.11–0.57; *p* = 0.001). Figure 1 summarizes the main findings from the univariate and multivariate analyses on clinical predictive factors of SBS and IF.

### 3.5. Time-Dependent Risk of SBS Development

**Figure 2:**
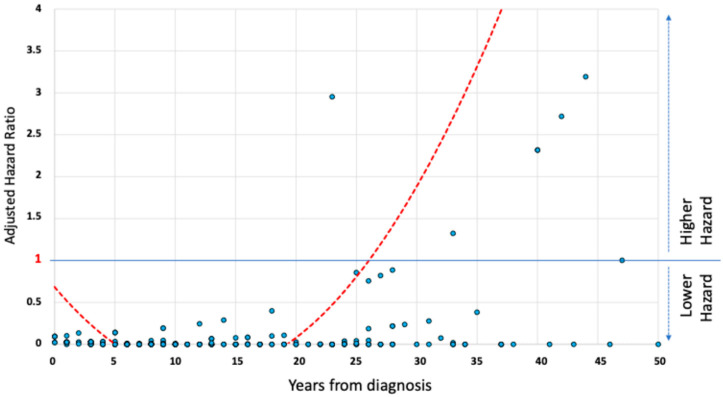
The relationship between adjusted hazard of short bowel syndrome development and disease duration in Crohn’s disease patients. A graphical scatterplot of the relationship between adjusted hazard of SBS development and duration of the disease in the study population. The trend curve shows an exponential increase in hazard after the third decade of disease, exceeding a hazard ratio of 1 after 26 years from the diagnosis of Crohn’s disease.

The relationship between disease duration and adjusted risk of developing SBS is represented by an exponential growth curve (Figure 2). Starting from the 26th year of disease, the adjusted risk exceeds the threshold of a hazard ratio (HR) of 1, reaching values greater than 2 around 30 years after diagnosis.

### 3.6. Predictive Accuracy of Surgical Parameters for SBS

**Figure 3:**
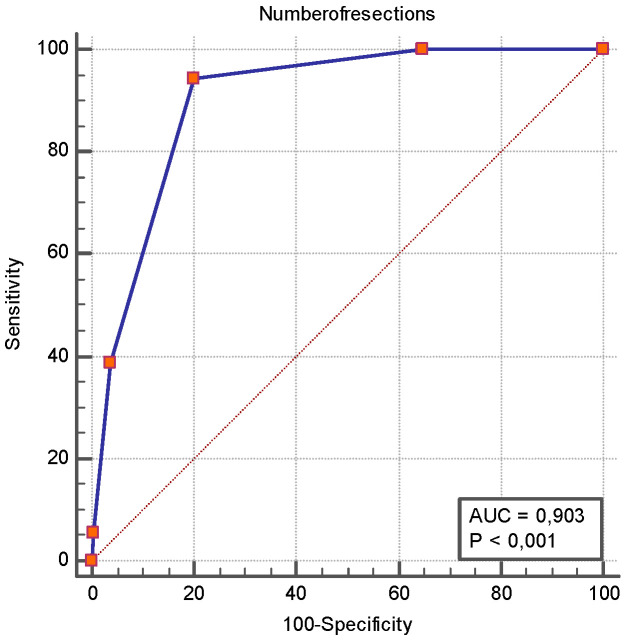
**ROC curve analysis of number of surgical resections as a predictor of short bowel syndrome**. The ROC analysis revealed that the number of surgical resections had an AUROC of 0.903 [0.857–0.938] for SBS, and a number of resections ≥ 2 had a sensitivity of 94.4% [72.7–99.9] and a specificity of 79.9% [73.9–85.1] for SBS occurrence.

**Figure 4:**
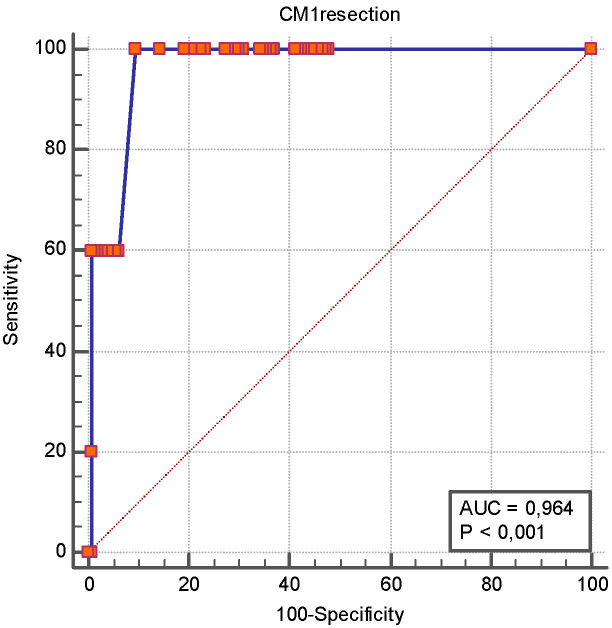
**ROC curve analysis of length of first intestinal resection as a predictor of short bowel syndrome.** The ROC analysis revealed that the length of the first intestinal resection had an AUROC of 0.964 [0.921–0.988] for SBS, and an intestinal resection of more than 50 cm had a sensitivity 100% [47.8–100.0] and a specificity of 90.4% [84.4–94.7] for SBS occurrence.

As reported previously, the total number of surgical resections was associated with the occurrence of SBS. Figure 3 shows the ROC analysis of the number of surgical resections as a predictor of SBS. Similarly, Figure 4 illustrates the ROC analysis of the length of the first intestinal resection as a predictor of SBS. Table 4 shows the 95% confidence intervals for sensitivity, specificity, positive and negative predictive values, and likelihood ratios at clinically realistic thresholds for the first resection greater than 50 cm.

## 4. Discussion

Our prospective study provides new insights into the key clinical predictors of SBS, marking an initial step toward translating these findings into prevention strategies. Consistent with the previous literature, we found that UGI (Montreal L4) and prolonged disease duration significantly increase the risk of developing SBS. The striking independent association of L4 (OR~20) highlights the aggressive behavior of proximal CD, which often necessitates extensive resections and postoperative complications [18]. More recent data indicate that proximal small-bowel lesions are associated with greater inflammatory burden and prompt initiation of biologic therapy even in mild disease, underscoring the clinical significance of proximal involvement in treatment stratification [19]. However, the UGI could be heterogeneous, with a more proximal phenotype associated with a better prognosis (esophagogastric duodenum involvement compared to jejunal or proximal ileal involvement) [20]. This means that the location of the disease must be carefully staged and monitored to identify patients at the highest risk of disease severity. The cumulative impact of long-term inflammation and repeated surgeries also aligns with prior data indicating that the risk of SBS typically rises after 15–20 years of disease [9,21]. Our time–risk curve qualitatively mirrors such observations. In fact, we show that the risk exceeds a HR of 1 around 25 years post-diagnosis and rises steeply thereafter, with a double-adjusted HR after 30 years of disease. A notable finding is that early exposure to biologics and advanced therapies significantly reduces the risk of SBS (OR~0.25). This finding is supported by a meta-analysis demonstrating that early biologic initiation (<3 years from diagnosis) reduces the odds of CD-related surgery (OR = 0.63) [22]. Advanced therapeutic agents are associated with mucosal healing or endoscopic improvement [23]. This, in turn, reduces the likelihood of progression to stricturing or penetrating phenotypes, which are more closely linked to surgical intervention [24]. Contemporary real-world and guideline-oriented evidence further supports early, treat-to-target use of advanced therapies to prevent structural damage [25]. In the postoperative setting, a recent multicenter study showed that a stratified risk-based biologic prophylaxis reduced endoscopic recurrence and subsequent adverse outcomes, underscoring the preventive role of advanced therapies after resection [26]. Additionally, several studies on postoperative recurrence similarly conclude that earlier/top-down biologic strategies can lower endoscopic recurrence and downstream complications [27,28]. Our results showed that exposure to a higher number of advanced therapies was independently associated with a lower risk of developing SBS. This finding challenges the assumption that sequential use of multiple advanced therapies necessarily reflects more refractory disease. Although it may seem unexpected, it suggests that exposure to advanced therapies is generally associated with a lower disease burden. An alternative explanation is that patients receiving multiple advanced treatments may have also achieved tighter disease control. The delayed initiation of biologics observed in our cohort of SBS patients may indicate underutilization of top-down strategies in real-world practice. However, this is likely attributable to their longer disease history, as many were diagnosed and initially treated in the biological era, or when the step-up approach was the prevailing evidence-based strategy.

Consistent with prior case–control studies, a higher number of resections and a longer initial intestinal resection were strongly predictive of SBS [8,29]. In our ROC analysis, ≥2 resections and a first resection length >50 cm achieved excellent discrimination (AUROC > 0.90). However, we must emphasize that our patient sample size is insufficient to provide definitive information; therefore, these results should be considered with caution. In other words, these thresholds may only serve to guide surgical decision-making, aiming to preserve bowel length and avoid repeated resections, which could potentially modify the subsequent risk trajectory of SBS.

Therefore, our data also support the use of bowel-sparing techniques to preserve small bowel, as well as multidisciplinary decision-making to minimize the extent of resections [2].

Although only a small proportion of patients in our cohort developed IF (9 patients), the characteristics of this subgroup are clinically meaningful. Patients with IF had a significantly younger age at diagnosis and a markedly longer disease duration compared to the rest of the cohort. This suggests that a longer inflammatory burden over time, particularly in early-onset disease, may play a critical role in the pathogenesis of IF. Consistently, the higher prevalence of UGI and fistulizing behavior in this subgroup reinforces the notion that extensive, transmural disease (particularly when proximally located) predisposes to severe malabsorption and functional loss of intestinal surface. Interestingly, while SBS is frequently a prerequisite for CIF occurring in patients with CD, we did not observe significant differences between SBS patients with and without IF, likely due to the small sample size. From a clinical perspective, these findings overall underscore the importance of early identification of patients at high risk of developing SBS (and CIF) to implement timely interventions, including nutritional support or referral to intestinal rehabilitation centers. This aligns with the 2023 ESPEN guideline update on CIF, which emphasizes early referral to specialized IF centers, multidisciplinary care pathways, and proactive nutritional strategies to optimize outcomes [17].

Our study has some limitations that must be considered when interpreting the results. The number of SBS and IF events was relatively limited; therefore, our sample size is underpowered to provide definitive insights. Moreover, our analysis did not account for certain potentially relevant risk factors, such as the cumulative amounts of systemic corticosteroids or budesonide therapy, which have been identified as independent predictors of SBS in previous studies [8]. Notably, a single-center design is prone to referral bias. Therefore, a causal relationship between biological exposure and lower SBS risk cannot be definitively established, as indication bias may influence who receives early biologic therapy. Also, confounding by disease severity, due to the partial availability of data on inflammatory markers throughout the disease course, cannot be entirely ruled out. Although we believe that assessing inflammatory markers at a single time point would not adequately capture the chronic burden of inflammation, we acknowledge the possibility of residual confounding by these parameters, as also emphasized in a previous study, which identified the cumulative burden of inflammation as a risk factor for SBS [9].

Interestingly, a new serological marker for assessing intestinal absorptive function appears promising: butyrylcholinesterase (BChE). Indeed, a study in patients with CD undergoing nutritional rehabilitation found that serum BChE levels increased significantly over time, mirroring improvements in weight, BMI, albumin, and hemoglobin [30]. Therefore, it is plausible that patients with CD and SBS-CIF could also be monitored using serial measurements of BChE levels. Therefore, further studies incorporating this marker would be desirable.

Conversely, the present study has several strengths. Firstly, the outpatient setting may more accurately reflect real-world clinical management of patients, thus supporting the early initiation of biologics/small molecules, particularly for patients with upper GI disease localization or early fistulizing features. Furthermore, we identified some independent predictors of SBS that, if further confirmed in a larger patient population, could be used to construct a risk stratification model. Incorporating Montreal L4 status, disease duration, and both the number and extent of resections into a prognostic model may enable the earlier identification of high-risk patients.

In conclusion, our findings highlight the importance of early identification of patients at risk for SBS and the subsequent implementation of timely and effective therapeutic strategies. Optimizing medical management through the early initiation of advanced therapies, together with the adoption of bowel-preserving surgical techniques, appears pivotal in mitigating the long-term risk of SBS. However, to ensure the robustness and generalizability of these results, multicenter studies in larger, more diverse cohorts or data from national (or multinational) SBS/IF patient registries are warranted to validate the identified predictors and refine clinically applicable risk thresholds. Furthermore, integrating imaging-based parameters and serological biomarkers into predictive algorithms may substantially enhance the precision of risk stratification models. In parallel, the health-economic dimension of proactive therapeutic strategies warrants careful consideration, particularly in light of recent modeling studies demonstrating the cost-effectiveness of earlier adoption of advanced therapies in CD [31]. Finally, interventional trials specifically designed to test early biologic initiation and bowel-sparing surgical approaches in high-risk patient subgroups are needed to determine whether such strategies can effectively prevent the progression to SBS and, ultimately, CIF.

## Figures and Tables

**Table 1:** Demographics and clinical characteristics of the cohort.

Demographics	*n* (232)	%
Male *n* (%)	122	52.6
Age Median (IQR)	49 (37–60)	
Montreal Classification		
A1	26	11.2
A2	146	62.9
A3	60	25.9
B1	60	25.9
B2	109	47
B3	63	27.1
L1	84	36.2
L2	23	9.9
L3	113	48.7
L4	12	5.2
Perianal Disease N (%)	56	24.1
Active Smoker	57	24.6
Surgery	157	67.7
Number of Surgeries in the Course of the Disease		
0	75	32.3
1	95	41
2	46	19.9
≥3	16	7.1
Extraintestinal Manifestations (Yes)	50	21.6
Short Bowel Syndrome	18	7.8
Actual Therapies		
Advanced Therapies	223	96.1
Infliximab	50	21.5
Adalimumab	61	26.3
Vedolizumab	28	12.1
Risankizumab	15	6.5
Ustekinumab	65	28
Upadacitinib	4	1.7
Conventional Therapy	9	3.9
Number of Different Advanced Therapies in the Course of the Disease		
0	9	3.9
1	97	41.9
2	69	29.6
≥3	57	24.6
Enteral Nutrition	6	2.6
Parenteral Nutrition	7	3
Revestive	2	1

**Table 2:** Demographic and clinical features in Crohn’s disease patients with and without SBS.

	CD (*n* = 214)	CD-SBS (*n* = 18)	*p*
Age, Median (IQR)	49 (37–59)	56.5 (39.5–65.5)	0.120
Male *n* (%)	112 (52.3)	10 (55.6)	0.793
BMI, Median (IQR)	23.5 (21.2–26.9)	22.3 (20.5–23.1)	0.028
Active Smoker	54 (25.6)	3 (16.7)	0.794
Montreal Classification:			0.123
A1	22 (10.3)	4 (22.2)	
A2 + A3	192 (89.7)	14 (77.8)	
L1 + L2 + L3	207 (96.8)	13 (72.2)	<0.001
L4	7 (3.2)	5 (27.8)	
B1 + B2	160 (74.8)	9 (50)	0.023
B3	54 (25.2)	9 (50)	
A1	22 (10.3)	4 (22.2)	0.123
A2 + A3	192 (89.7)	14 (77.8)	
Perianal Disease, *n* (%)	48 (22.4)	8 (44.4)	0.036
Extraintestinal Manifestations, *n* (%)	48 (22.4)	2 (11.1)	0.262
Duration of Disease (Years), Median (IQR)	10 (6–22)	27 (16.75–40.25)	<0.001
Total Count of Advanced Therapies, n (%)			0.338
0	9 (4.2)	0	
1	92 (43)	5 (27.8)	
2	64 (29.9)	5 (27.8)	
≥3	49 (22.9)	8 (44.4)	
Age at First Surgery, Median (IQR) *	37.5 (27.8–49)	31.5 (24.5–35.5)	0.070
Extension of First Resection (cm), Median (IQR) *	30 (16–50)	100 (60–110)	<0.001
Advanced Therapy After First Resection *	118 (84.9)	8 (44.4)	<0.001
Years from Diagnosis to First Advanced Therapy, Median (IQR)	3.5 (1.0–11.0)	16.0 (8.75–26.5)	<0.001

* Variables apply only to patients who underwent bowel resection (not all patients underwent bowel resection).

**Table 3:** Multivariate logistic regression. Data are presented as odds ratios (ORs) with 95% confidence intervals (95% CIs) and *p*-values (*p*).

Variable	OR	95% CI	*p*
Duration of disease	1.083	1.025–1.145	0.005
Montreal L4	20.079	2.473–163.06	0.005
Montreal B3	0.903	0.243–3.359	0.879
Perianal disease	2.199	0.593–8.160	0.239
Number of advanced therapies until SBS	0.247	0.107–0.58	0.001

**Table 4:** Impact of first intestinal resection extension on SBS development. Table shows 95% confidence intervals for sensitivity, specificity, PPV, and NPV of first resection extension. Abbreviations: CI: confidence interval; PPV: positive predictive value; NPV: negative predictive value.

Resection Length	Sensitivity	95% CI	Specificity	95% CI	PPV	95% CI	NPV	95% CI
≥0 cm	100.00	47.8–100.0	0.00	0.0–2.5	3.3	3.3–3.3	-	-
>50 cm	100.00	47.8–100.0	90.41	84.4–94.7	26.3	17.8–37.0	100.0	-
>60 cm	60.00	14.7–94.7	93.84	88.6–97.1	25.0	11.4–46.4	98.6	95.9–99.5
>95 cm	60.00	14.7–94.7	99.32	96.2–100.0	75.0	27.3–96.0	98.6	96.1–99.5
>120 cm	0.00	0.0–52.2	99.32	96.2–100.0	0.0	-	96.7	96.6–96.7
>150 cm	0.00	0.0–52.2	100.00	97.5–100.0	-	-	96.7	96.7–96.7

## Data Availability

The data supporting the findings of this study are available upon request from the corresponding author.

## Abbreviations

The following abbreviations are used in this manuscript:

AUROC	Area Under the ROC Curve
CD	Crohn’s Disease
CIF	Chronic Intestinal Failure
CI	Confidence Interval
EIM	Extraintestinal Manifestation
GLP-2	Glucagon-Like Peptide-2
HR	Hazard Ratio
IBD	Inflammatory Bowel Disease
IF	Intestinal Failure
OR	Odds Ratio
ROC	Receiver Operating Characteristic
SBS	Short Bowel Syndrome
TNF-α	Tumor Necrosis Factor-Alpha
